# Structure–activity features of purines and their receptors: implications in cell physiopathology

**DOI:** 10.1186/s43556-022-00068-1

**Published:** 2022-01-26

**Authors:** Mauricio Díaz-Muñoz, Rolando Hernández-Muñoz, Armando Butanda-Ochoa

**Affiliations:** 1 Departamento de Neurobiología Celular Y Molecular, Instituto de Neurobiología, Universidad Nacional Autónoma de México, UNAM, Boulevard Juriquilla 3001, C.P. 76230 Juriquilla, Querétaro México; 2 Departamento de Biología Celular Y Desarrollo, Instituto de Fisiología Celular, Universidad Nacional Autónoma de México, UNAM, Ciudad Universitaria/Circuito Exterior, C.P. 04510 Ciudad de México, México

**Keywords:** Purines, ATP, Adenosine, ADORA, Cancer, Hepatocarcinoma

## Abstract

The purine molecular structure consists of fused pyrimidine and imidazole rings. Purines are main pieces that conform the structure of nucleic acids which rule the inheritance processes. Purines also work as metabolic intermediates in different cell functions and as messengers in the signaling pathways throughout cellular communication. Purines, mainly ATP and adenosine (ADO), perform their functional and pharmacological properties because of their structural/chemical characteristics that make them either targets of mutagenesis, mother frameworks for designing molecules with controlled effects (e.g. anti-cancer), or chemical donors (e.g., of methyl groups, which represent a potential chemoprotective action against cancer). Purines functions also come from their effect on specific receptors, channel-linked and G-protein coupled for ATP, and exclusively G-coupled receptors for ADO (also known as ADORAs), which are involved in cell signaling pathways, there, purines work as chemical messengers with autocrine, paracrine, and endocrine actions that regulate cell metabolism and immune response in tumor progression which depends on the receptor types involved in these signals. Purines also have antioxidant and anti-inflammatory properties and participate in the cell energy homeostasis. Therefore, purine physiology is important for a variety of functions relevant to cellular health; thus, when these molecules present a homeostatic imbalance, the stability and survival of the cellular systems become compromised.

## Introduction

The purine molecule is a basic structural frame that results from the fusion of a pyrimidine ring with an imidazole one. This ring system may adopt any of four (most stable) N–H tautomeric forms whose stability depends on the aromaticity of the ring system [1]. In adenine, tautomers interconvert from amine to imine, and in guanine the tautomeric transition implies interconversion between keto form to enol form [2, 3]. This transition between tautomers comes from either prototropy involving an extranuclear atom (e.g. form water molecule), or by prototropy involving only ring atoms [4–6]. Within nucleic acids, specially DNA, these tautomeric forms are main structural pieces in the dynamics of the inheritance processes and the interconversion of the different adenine or guanine tautomers causes mispairing with pyrimidines bases, leading to changes in the sequence of nucleic acids (genetic mutations) so for example in adenine the imine form may cause spontaneous mutagenesis since it does not couple with thymine in the DNA double helix [2, 7]. In the case of guanine, this purine is the most frequent involved in mutagenesis and cancer since some carcinogens like aflatoxin B_1_ bind to it [8]. Nevertheless, there are also some anti-cancer drugs like cis-platin that bind to guanine too [9].

Near a century ago, in 1929, Drury and Szent-Gyorgyi reported [10] cardiac actions for adenine compounds in mammalian experimental systems. After decades, Burnstock in 1978 [11] had the conceptual capacity to propose a cellular communication system based in the recognition between purinergic ligands and their corresponding receptors. Because of these initial observations, most of the studies were focused on the physiological actions of purines on brain and heart cells [12]. In this context, the first reports regarding pathological consequences on altered purinergic transmission were rapidly reported, particularly, the role that released ATP from epithelial cells, platelets and sympathetic nerves, plays in the development of intimal thickening during arterial diseases, such as arteriosclerosis and restenosis after angioplasty, and in the growth of new vessels that takes place during wound healing and in tumors [13]. Therefore, other biological roles of purines that are discussed along this work involve the participation of these molecules as messengers that modulate the function of their receptors in intracellular signaling pathways affecting functions such as the expression of tissue markers, the secretion of insulin, the anti-inflammatory process, etc. Nevertheless, purine signaling pathways are also involved in tumor growth and progression [14], thus, purine ring system represents an important mother structural frame to design derivatives with anti-cancer activity which has been achieved mainly by chemically modifying the purine ring system of adenine either alone or bound to a ribose molecule forming the nucleoside known as adenosine (ADO), where the modification of the monosaccharide also influences the anti-cancer activity.

**Fig. 1:**
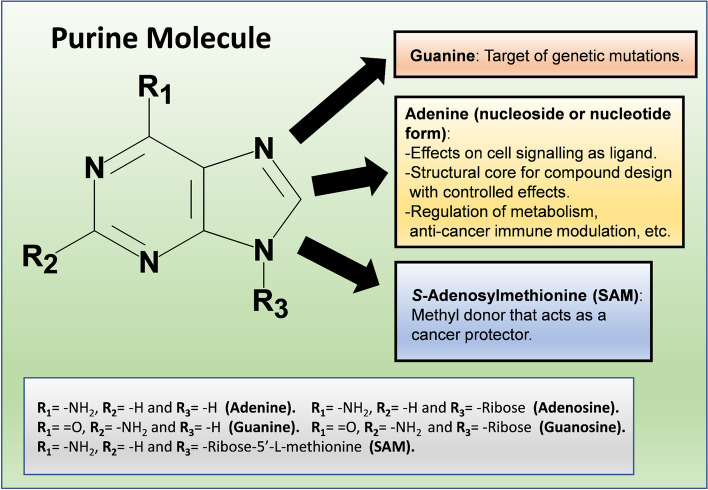
Summary of purines structure and cellular function

Besides the anterior, it is also discussed the role of purines as metabolic intermediaries such as S-Adenosylmethionine (SAM) that intervenes in liver protection against alcohol toxicity or as a chemoprotective methyl donor against liver cancer. Therefore, an adequate function of the whole purinergic physiological system is required to preserve cell life and health, and any deviation from homeostasis compromises cell stability. In this review, the metabolic and cell signaling aspects of purines are considered in an integrative manner with some purine structural features and their cellular receptors which are relevant in cell physiology and disease (e.g. cancer, particularly hepatocellular carcinoma) (Fig. 1).

## Nucleotide/nucleoside-based cellular signaling

**Fig. 2:**
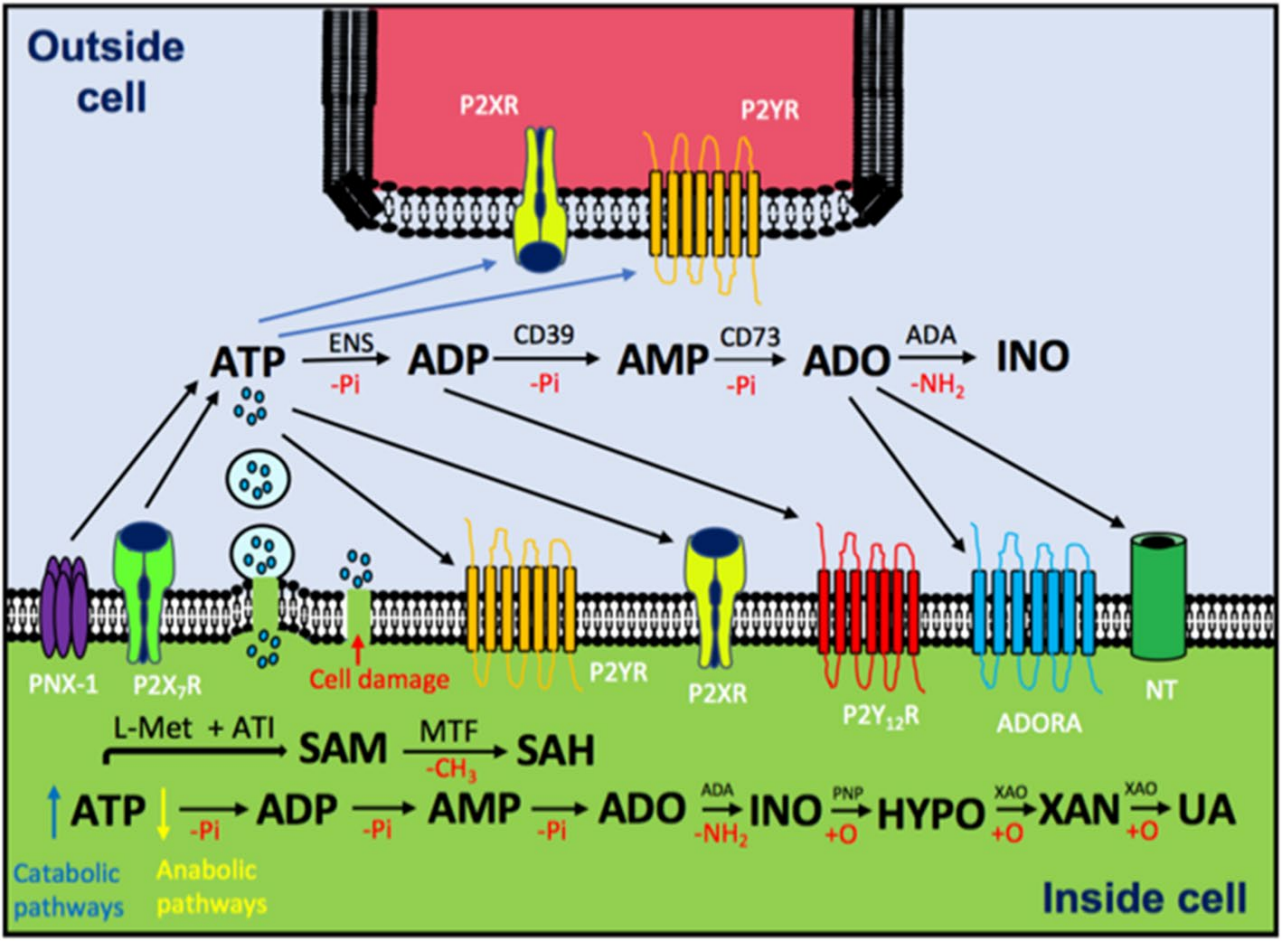
Schematic representation of purinergic communication and metabolism. ATP levels inside the cells are reduced by anabolic pathways or (depending on energy charge) incremented by catabolic pathways (e.g. glycolysis and oxidative phosphorylation in the mitochondria), such ATP is released to the extracellular space by exocytosis, transporters, hemichannels, pannexin-1 (PNX-1), P2X_7_R or simple cell membrane disruption due to cell damage (e.g. caused by cancer) In the extracellular space, ATP stimulates P2XR (ligand-activated ion channels), P2YR (G-protein coupled receptors) or it can be hydrolyzed, which means one inorganic phosphate less (-Pi), by CD39 and CD73 (ecto-nucleotidases, ENS) to ADP, AMP and ADO. In particular, ADP stimulates P2Y_12_R and ADO modulates ADORAs. The nucleoside can be deaminated by adenosine deaminase (ADA) to produce inosine (INO) or introduced into the cell by nucleoside transporter (NT). Within the cell, SAM is synthetized from L-methionine and ATP by a reaction catalyzed by adenosyl transferase isoenzymes (ATI) and with the help of Methyl Transferases (MTF) it is converted to SAH. The catabolism of ADO goes from deamination (to obtain inosine) catalyzed by ADA to consecutive oxidations (+ O) to obtain hypoxanthine (HYPO) (reaction catalyzed by purine-nucleoside phosphorylase, PNP), Xanthine (XAN) and finally Uric Acid (UA), both reactions catalyzed by xanthine oxidase (XAO)

Almost fifty years ago, Dr. Geoffrey Burnstock postulated a form of cellular communication based in purinergic molecules [15]. Soon after, researchers found that pyrimidine molecules (UTP, UDP and UDP with sugars attached) were also involved in this phenomenon, in addition to ATP and ADO [16]. This cellular communication is possible because nucleotides and nucleosides are recognized by specific membrane receptors. These ubiquitous receptors belong to two families: G protein-coupled receptors (GPCRs) and ligand-gated ion channels (LGICs) [17]. Adenine base has also been reported as an endogenous ligand for a specific receptor, which is a different type for ATP and ADO receptors (ADORAS) [18] (Fig. 2). Purinergic signaling is present along the phylogenetic scale, which is under circadian regulation and has an ubiquitous distribution on tissues and organs explored so far. Indeed, various physiological functions have been associated with the biological roles of purines, such as regulation of the immune response, sleep–wake cycle, vasodilation, lung activity, and many others [19].

ATP receptors, also known as P2 receptors, are classified into two groups: P2X, which are trimeric LGICs, and P2Y receptors, which are GPCRs. The P2X receptors are cationic ion channels that can be formed by seven different subunits to act as homo- or heterotrimers. On the other hand, P2Y receptors consist of seven membrane-spanning transmembrane helices that interact with Gs, Gq or Gi/0 proteins. They form a subgroup that consists of eight different receptors. ADO is also a ligand for purinergic receptors known as ADORAs (previously named P1 receptors). All four recognized ADORAs are GPCRs transducing by Gs, Gi/0 and Gq. It has been shown that some ADORAs can form heteroreceptors by interacting with dopamine and glutamate GPCRs [20]. There are several reviews regarding the physiological and pathological roles that ATP and ADO receptors play in different tissues, organs and species [21–23]. Likewise, the pharmacology of most of these receptors is greatly developed, since it has been the subject of systematic academic efforts and innovative entrepreneurial interests [24, 25].

Interestingly, due to the dynamic and fine regulated transformation of ATP into ADO (according to the presence of a diverse enzyme with ecto-nucleotidase activity), in purinergic signaling pathways exists an interrelation between the action of ATP with the action of ADO, sometimes such interrelation results to be complementary and some others is antagonistic. Purine-dependent signaling in a given cellular system results from the presence of ATP receptors and ADORAs, as well as the proportion of ATP and ADO acting as extracellular ligands [26]. Another feature that makes more interesting the signaling role played by purines is the existence of an intracellular metabolic network underlying the cellular bioenergetics that is also dependent on the proportion of phosphorylated and non-phosphorylated purines (from ATP to ADO, with ADP and AMP as mediators). This situation supports the notion of “purinome,” as the set of coordinated actions among intracellular and extracellular purines as a key element to regulate cellular activity [27]. In the next sections, it is presented how the action of ADO makes the “purinome” works.

### ADO actions mediated through its receptors

ADO and 2-chloroadenosine (a metabolically stable analogue), are able to reduce matrix mineralization and down regulate the expression of bone tissue markers by activating a non-specific alkaline phosphatase (TNAP) in human aortic smooth muscle cells (HASMCs), without affecting other osteoblastic differentiation markers (e.g. Runt-related transcription, factor 2 or Bone Sialoprotein II). This effect is mediated by ADORA3, since its knockdown reverses the effects promoted by these two molecules, and its specific stimulation with IB-MECA (a selective ADORA3 agonist) reduces matrix mineralization and the expression of TNAP [28].

ADORA3 may also represent a potential therapeutic target in the treatment of Diabetic Nephropathy, since its selective antagonism reduces the levels of the pro-fibrotic marker alpha smooth muscle actin (α-SMA) and attenuates the levels of inflammatory mediators such as IL-1β and IL-18, (and others: IL-1 IL-1β, IL-18, IL-6, and IL-10), by avoiding the increment of caspase 1 and the nuclear localization of the nuclear factor kappa B (NFκB) into the nucleus of the renal tubular epithelium cells in diabetic rats [29].

### Antioxidant and anti-inflammatory properties of ADO

The absence of ADORA2A in primary murine chondrocytes promoted spontaneous osteoarthritis with concomitant mitochondrial damage (swelling, reduced mitochondrial content and dysfunction) and ROS-induced oxidative damage. Treatment of IL-1β-stimulated murine chondrocytes with CGS21680, an ADORA2A agonist, increased mitochondrial content, improved mitochondrial function and reduced ROS production. This agonist induced ADORA2A effect was also observed in human chondrocytes, so that ADORA2A signaling seems to be ligated to mitochondrial and oxidative protection in osteoarthritis [30].

On the other hand, the transcriptional modulation of tissue levels of NFkB, tumor necrosis factor alpha (TNF-α), plasmatic levels of pentraxin-3 as well as lipid peroxidation in rats, are related to the anti-inflammatory and healing effects of ADO in the inflamed colon tissue; so apparently this nucleotide promotes the healing of colon and rectum (in acetic acid-induced acute colitis) through an immunosuppressive effect in the intestinal tissue that contributes to heal ulcerative colitis [31].

### Expression of ADORAs and insulin secretion

Immunohistochemical studies showed that ADORA2A and ADORA2B are expressed in the luminal membranes of rat and human pancreatic duct cell lines. Additionally, ADORA2A was detected in the rat islets, most likely in β cells [32]. ADORA1 and ADORA2A were also found in isolated α-cells from mouse islets, and the use of an ADORA2A agonist induced a stimulation of glucagon secretion [33]. Findings indicate that ADO regulates pancreatic exocrine and endocrine secretions through the acini-to-duct signaling in the exocrine pancreas. ADORA2A co-localized with ezrin (vilin-2), a linker between plasma membrane and actin cytoskeleton, in the luminal membrane of pancreatic duct cells in mouse and guinea pig, and ADORA2B was detected in insulin-positive β cells in islets of Langerhans. Also, ADORA2A may be partly involved in the exocrine secretion of pancreatic duct cells through acini-to-duct signaling [34].

It was shown that a non-peptidyl small molecule, ADO,5′-Se-methyl-5′-seleno-,2′,3′-diacetate can restore insulin resistance as evidenced by biochemical parameters such as blood glucose levels and the determination of active signaling molecules by western blot analysis, concluding that ADO,5′-Se-methyl-5′-seleno-,2′,3′-diacetate administration could restore insulin receptor activation, even in the absence of the pancreatic hormone; this modified nucleoside significantly promotes circulating glucose clearance. It was found that the compound activates the response of insulin receptor in liver and skeletal muscle cells, reduces the expression of the catalytic subunit of glucose-6-phosphatase by cooperating with insulin, and stimulates glucose uptake. Moreover, the direct interaction of the compound with the insulin receptor α (INSRα) triggers its activation (by phosphorylation) and its downstream targets, but without activating the insulin-like growth factor 1 receptor in the liver and the in skeletal muscle. Apparently, this modified nucleoside could be used as an insulin replacement therapy in diabetes as well as in other diseases associated with insulin resistance [35].

### Purine metabolism and receptor-mediated signaling in tumor growth

Purinergic signaling also constitutes an important physio-pathological component that confers an invasive potential to tumor cells. For example, as reviewed by Braganhol et al. 2020 [36], the alterations of purinergic signaling are involved in the progression of these tumors in neural tissues. In this sense, gliomas have a low expression of ecto-nucleotidases compared to astrocytes in culture so nucleotides can induce glioma proliferation [37]. The importance of extracellular ATP for glioma pathobiology was confirmed by degrading it to AMP, which reduced the size of neural tumors. In comparison, an increase in tumor size was induced by over-expression of an unspecified ecto-enzyme that degrades ATP to ADP, indicating that extracellular ATP metabolism plays a role in tumor growth [38]. This ATP effect additionally involves the purinergic receptors on glioma progression, particularly the P2X7 receptor, which can participate in the resistance to ATP-induced cell death [39].

Besides, in vitro studies, have shown that treatment with 100 μM ATP or ADO, induce proliferation in U251-Mg, U138-MG and U-87 MG human glioma cell lines, exhibiting also an increase of the transport of thymidine into the cell, suggesting a role for these molecules beyond the sole regulation of the cell cycle [40], In U-138 MG cells such proliferative stimulation was also observed for 100 μM of ADP, UTP, inosine and guanosine probably through the participation of the P2Y_4_ and ADORA3 receptors.

As mentioned before, ATP (as well as ADO) activates the proliferative ERK, MAPK and PI3K/Akt pathways in U138MG human glioma cells. Now, considering the hypoxia that commonly occurs inside and around the tumor, it seems probable that the release of ATP and ADO to the extracellular space after cell lysis provides a favorable environment for tumor growth through the activation of both proliferative and anti-apoptotic pathways [41]. In glioma cells lines, ATP fails to induced cell death even at high 5 mM concentration [42], so apparently this resistance for ATP-induced cell death in glioma systems could be associated to a lack and/or to inactivation of the P2X7 receptor [43, 44]. Therefore, purinergic signaling may be considered as a future target for pharmacological treatments and even gene therapy.

On the other hand, the important role of ecto-nucleotidases has also been explored in bladder cancer systems, in both complete organism [45] and in cell lines [46]. In chemical-induced transitional bladder carcinoma, a weak immunostaining to NPDase3, and an elevated presence of CD73 were detected, suggesting that CD73 could be considered a marker for aggressive bladder cancer [45]. Consistently, in RT4, a cell line showing a low grade of invasiveness, the hydrolysis of ATP and ADP was more active than the hydrolysis of AMP. However, in T24 cell line, which shows a more aggressive cancerous profile, presented the opposite profile [46].

The incidence of purines and the purinergic pathways in pathophysiological processes, is also manifested by the action of some purine derivatives with physiological and pharmacological affects; for example, the 3-methyladenine displays an inhibitory effect on cell autophagy, which is a tightly regulated and a highly inducible process that it is believed to may be involved in the pathogenesis of some human diseases [47] since for example, it has been demonstrated that autophagy is involved in drug/chemical-induced liver injuries [48]. In this context, 3-methyladenine significantly reduces chloroform hepatotoxicity in mice, but acting via the activation of autophagy. These findings provide evidence that purines can be involved in autophagy but at the same time the use of 3-methyladenine to modulate autophagy in vivo demands caution [49]. In this sense, autophagy is critical for maintaining cellular homeostasis and its dysregulation has implications in health as well as in disease, for instance in cancer it has a dual role: as an inhibitor in tumor initiation but as pro-tumoral factor in cancer progression too [50].

### Crystallized structure of purinergic receptors

Now, since purinergic signaling involves the recognition of specific receptors, some structural aspects of those macromolecules should be considered to understand their physiological role furtherly. Next, we discuss the three-dimensional structure of five purinergic receptors:

#### P2X_7_ receptor

This is a very well characterized ion channel-linked purinergic receptor that was cloned at the end of the twentieth century. The mammalian receptor presents around of 594–5 amino acid residues in length. It functions as a trimer with each monomer containing 2 transmembrane domains, N- and C-termini located intracellularly, and an extensive extracellular ectodomain with 2 ATP-binding sites [51]. This receptor forms homotrimeric structures, but it has been reported heteromeric complexes with P2X_2_, P2X_4_ and P2X_5_ subunits [52]. P2X7 receptor is expressed in a large variety of organs and in many species, and it has been associated with inflammatory responses as well as cellular death [53]. 

The closed state of the trimeric P2X_7_ receptor from the giant panda was crystalized in 2016 [54]. It showed a very similar structure in comparison to the zebrafish P2X_4_ receptor: the large ectodomain contained 14 β-strands; there are 2 transmembrane α-helices, being the central one the forming pore structure. N- and C-termini remain to be structurally resolved being important in the gating of the channel as well as in the desensitization process. 

#### P2Y_12_ receptor

This receptor is abundantly expressed in platelets and is responsive to ADP. It has been reported that some alterations in the gene P2YR_12_ are associated with reduction of platelet function [55]. This receptor, by acting through a Gi protein, promotes inhibition in cAMP levels and activation of phosphatidylinositol-3-kinase and Rap1b proteins. Zhang K and Zhang J reported the structure of the P2Y_12_ receptor using high-resolution X-ray crystallographic studies in the presence of both a pharmacological agonist (2-methylthio-adenosine-5´-diphosphate) and an antagonist (AZD1283) [56, 57]. It was confirmed that the P2Y_12_ receptor belongs to the δ-branch of class A GPCRs, with the peculiarity that it showed an unusual straight α–helical conformation in TM5. Moreover, two binding sub-pockets were detected in the antagonist bound P2Y_12_ receptor [57]. Upon agonist binding, the P2Y_12_ receptor shows notorious rearrangements, especially the contraction of sub-pocket 2 [56]. Stabilization of the inactive P2Y_12_ receptor by charged residues located in the intracellular loops of TM3 and TM6, detected in several rhodopsin-like GPCRs, was not observed in the P2Y_12_ receptor [58]. 

#### P2 Y_1_ receptor

Receptor is also highly expressed in platelets and is the target of several antithrombotic drugs. In this case, ADP and ATP can act as ligands, and the signaling mechanism of the P2Y_1_ receptor is usually by intracellular calcium mobilization via the Gq protein. Zhang D et al. (2015) reported the solved X-ray crystal structure of the human P2Y_1_ receptor bound to two different antagonists (MRS2500 and BPTU) [59]. Both P2Y_1_ and P2Y_12_ receptors are located in platelets and targets of antithrombotic drugs, but they show significant structural differences. Distinct from all other known class A GPCRs, the P2Y_1_ receptor has a basic histidine residue at position 3.49 (HR), whereas the conserved motif is D/ER [60]. Therefore, in comparison to the P2Y_12_ receptor, the P2Y_1_ receptor in this position exerts a repelling action that results in a more extended side-chain conformation, stabilizing the C-terminus in a unique conformation. The P2Y_1_ receptor structure lacks helix VIII, and its two crystals, one with an agonist bound and another with an antagonist bound to it, are structurally distinct, mainly in helixes V, VI and VII. Another important difference is the shift of the extracellular end of helix III away from the axis of the 7TM helical bundle in the P2Y_1_ receptor compared to the P2Y_12_ receptor [59].

#### P2Y_14_ receptor

 This receptor is located in most tissues and organs, and is crucial in inflammatory processes including diabetes. It recognizes UDP and sugar derivatives of UDP as activators, inhibiting the production of cAMP as its principal signaling mechanism [61]. Although the crystal structure of the P2Y_14_ receptor has not been elucidated, Lu et al. (2019) have postulated new antagonists of this receptor by using homology modeling based on the three-dimensional structure of the P2Y_12_ receptor [62]. This approach has allowed researchers to propose more refined phenyltriazole structures, test the effects of different pharmacophores and develop the structure–activity relationship (SAR) of new antagonists for the P2Y_14_ receptor.

#### Adenosine receptors

In *Adenosiland*, a virtual space launched in 2012, sequence and structural information assigned to all cloned adenosine receptors is simultaneously analyzed. Using bioinformatics and chemoinformatics resources, *Adenosiland* provides several structure-based and ligand-based query functions to facilitate the exploration of adenosine receptor structures by extrapolating from primary sequences to three-dimensional architectures [63]. So far, very few crystallographic or three-dimensional information that describes ADORAs structure has been published. In 2012, Wei et al. published a study suggesting three-dimensional pharmacophore models for selective ADORA2A and ADORA2B antagonists [64]. Both pharmacophore models were validated toward several structurally diverse selective antagonists. The pharmacophore model for ADORA2A contained four chemical features: one aromatic ring, one positively ionizable element, one hydrogen bond acceptor lipid and hydrophobic moiety. For ADORA2B, the model contained one aromatic ring moiety, one hydrophobic aliphatic element and two hydrogen bond acceptor lipid features.

### Structure–activity relationship (SAR) approaches

The field of docking and molecular dynamics simulation with computer-assisted strategies based on the crystal structure and homology models of purinergic receptors has been useful to rationalize the specificity and potency of pharmacological ligands [65]. In this context, the functional and structural characterization of an allosteric binding site in the P2X_4_ receptor for the non-competitive blocker BX430 was reported. This information was extrapolated to other P2X receptors [66], taking advantage of the high-resolution crystallographic data available for the zebrafish P2X_4_ receptor to use molecular dynamics simulation to model the docking of the antagonist on a binding site located in the ecto-domain of this purinergic receptor.

Additionally, Tobinaga et al., reported new P2X_3_ receptor antagonists with a pyrrolinone skeleton [67]. This group reported that substituting a lead compound in a given position (R-24) showed a more effective and orally bioavailable molecule with an analgesic effect against neuropathic pain. Furthermore, SAR studies applied to develop a radioligand for brain ADORA1 allowed the synthesis of a blood–brain barrier permeable compound that was suitable for PET analysis. This partial agonist was synthetized from a non-nucleoside (methylpyridin-methyl(thio)pyridine) template with chemical substitutions to incorporate ^11^C within its molecule [68]. ADORA2 has been of great interest because of its physiological and pharmacological significance. Using *in-silico* studies and molecular dynamics simulation, researchers have discovered different structures of this receptor and pharmacological agents acting as novel agonists and antagonists. Other approaches used to understand the ADORA2 structure and function have involved virtual screening/docking and QSAR-based pharmacophore modeling [69].

## ADO metabolism

As mentioned before, the role of purines in cell signaling is interconnected with the metabolic network that maintains the cellular bioenergetics according to the proportion of phosphorylated and non-phosphorylated purines that goes from ATP to ADO, with ADP and AMP as mediators. ADO is a purine nucleoside found inside and outside the cells, acting as a chemical messenger with autocrine, paracrine, and endocrine actions as well as a metabolic intermediary. When injected intraperitoneally, ADO increases the energy charge of the adenylate system in the liver, causing an enhanced glycogen biosynthesis and diminished fatty acid catabolism, which confirms the regulatory role of the adenylate system in the cell [70]. In the liver ADO is mainly formed by de novo purine synthesis, adenine nucleotide phospho-hydrolysis, and hydrolysis of SAM. Extracellular ADO can exert its function through the activation of adenosine receptors (ADORA1, 2A, 2B, and 3), or it can be transported into the cells by the nucleoside transporter or ADO can also be deaminated to inosine by adenosine deaminase (either extracellularly or intracellularly) and finally catabolized to uric acid. The physiological actions of adenosine include modulation of the immune response, regulation of the metabolism, as well as modulation and maintenance of the energetic homeostasis of the tissues such as blood cells, liver, and brain [71, 72]. Thereby, ADO can be generated intracellularly after S-adenosyl homocysteine (SAH) hydrolysis or after ATP hydrolysis through extracellular ecto-nucleotidases, and then internalized to the internal milieu by specific transporters. Within the cell, ADO is phosphorylated by adenosine kinase, deaminated to inosine by adenosine deaminase, or converted to SAH by the reversed action of its corresponding hydrolase. Its metabolism is extensive, resulting in a short half-life of the administered nucleoside. In the extracellular space, low physiological ADO concentrations can activate the ADORA1 and ADORA2A subtypes, whereas high μM concentrations are needed to recruit the ADORA2B and ADORA3 subtypes. The ADORA1 and ADORA3 subtypes are either coupled to Gi proteins, which negatively regulate adenylyl cyclase activity, or Gq proteins [73–76] leading to the hydrolysis of phosphatidylinositol to generate inositol-1,4,5-trisphosphate (IP3) and diacylglycerol (DAG). IP3, in turn, activates its receptors in the endoplasmic reticulum and stimulates the calcium release from the intracellular stores, whereas DAG can activate protein kinase C (PKC) and additional second messenger pathways. Moreover, the ADORA3 subtype can also modulate various intracellular signaling pathways such as the Wnt and the NF-κB systems [77]. These receptor-mediated and metabolic properties of ADO allow diverse pharmacological actions of the nucleoside, which can seem contradictory under certain circumstances.

### ADO and SAM

SAM minimizes alcohol hepatotoxicity, which seems to be related to the prevention of mitochondrial proteome alterations. SAM administration preserved mitochondrial respiration in ethanol-fed rats; however, the mitochondrial levels of SAM were increased by ethanol and SAM treatment, which promoted multiple changes in the proteome of liver mitochondria. Nevertheless, the maintenance of relevant proteins involved in mitochondrial pathways for energy conservation and biosynthesis are mediators in the protective effects of SAM against alcohol toxicity [78]. In fact, SAM has been used as an anti-inflammatory analgesic to treat depression and rheumatoid arthritis. Its modulating effect on jaundice related to chronic hepatitis B has also been shown in patients with mutations in the gene of glycine N-methyltransferase who show impaired SAM metabolism [79]. Several experimental models in animals during preclinical trials have shown a clear beneficial effect from methionine and SAM to prevent and treat these liver diseases [80].

### Participation of ADO salvage in oocyte energy homeostasis

The ADO salvage pathway involves two steps: First, AMP is phosphorylated to ADP and then ADP to ATP, this might be an alternative pathway for ATP production to satisfy the energetic demands in the meiotic maturation of the oocyte, therefore the ADO salvage pathway has to be active in order to take advantage of the large amount of AMP generated by phosphodiesterase breakdown of cAMP [81, 82]. In fact, it has been found that ATP is generated from AMP through the adenosine salvage pathway in mouse oocytes, and that the increment in the cAMP levels modifies adenine nucleotide metabolism, providing the AMP required for energy production through this salvage pathway [83].

## Purines in the context of cancer

### Purines and mutations

**Fig. 3:**
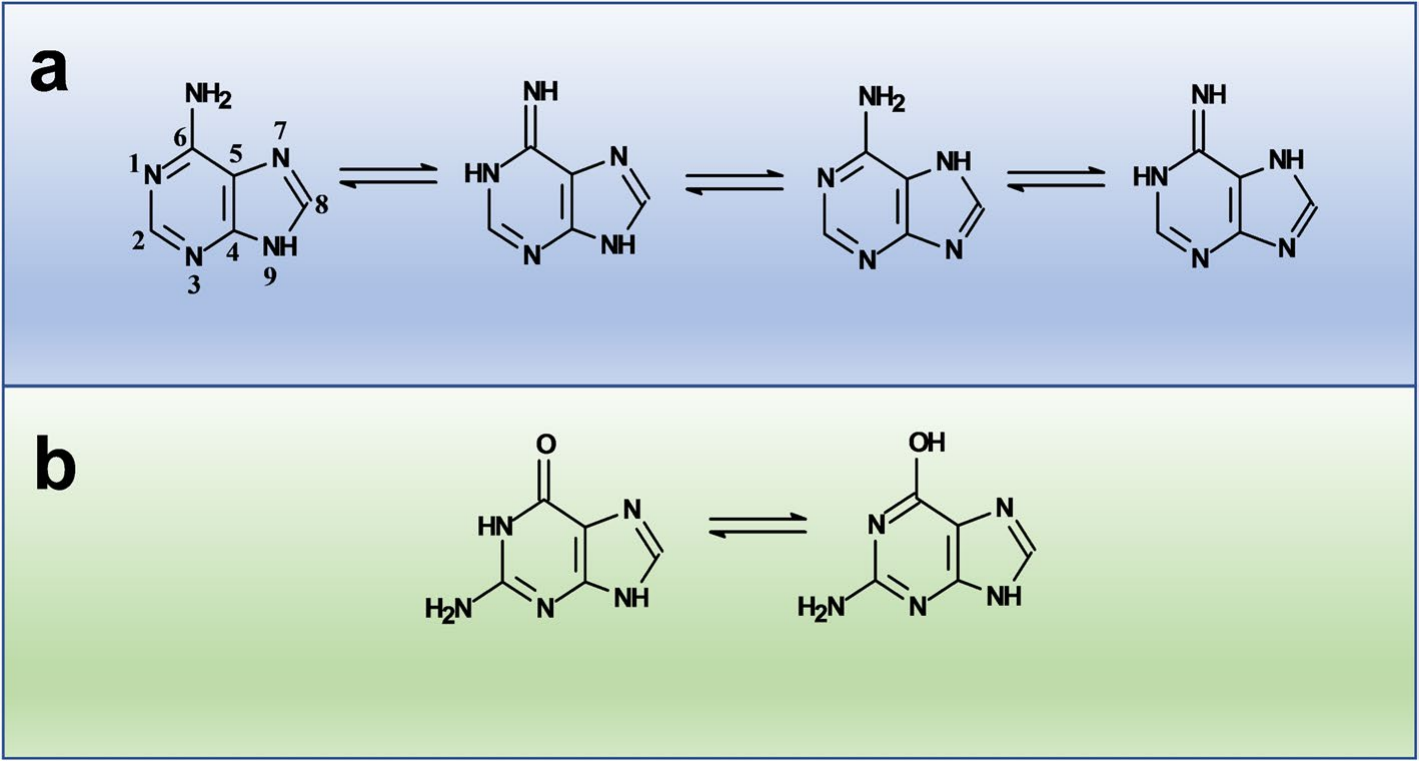
Adenine and Guanine tautomers in DNA. a) Amino-imino tautomers of Adenine are the result from the location of the hydrogen atom at N(7) and N(9) (see [2, 6] for stability details). b) Guanine keto-enol tautomers result when keto (> C = O) substituent (attached to C6 of guanidine) is protonated and transits to a hydroxyl (-OH) group. Note that the N(1) changes from amino to imino tautomer as well

**Fig. 4:**
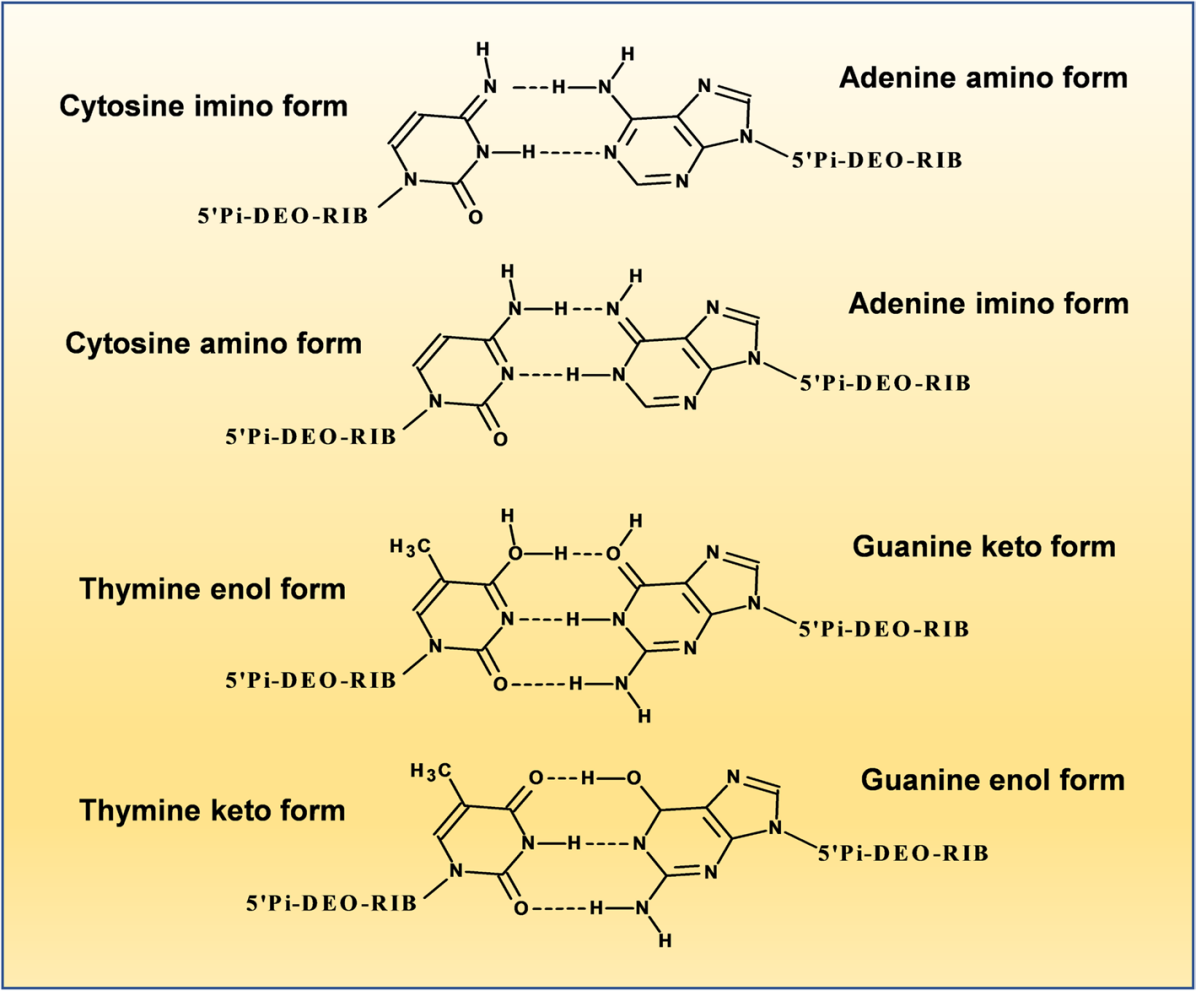
Pairing of Adenine and Guanine tautomers in DNA. Adenine (amino-imino) and Guanine (keto-enol) tautomers paring with tautomeric forms of Cytosine (amino-imino) and Thymidine (keto-enol), respectively (based and modified from [85])

As previously mentioned, the purine molecule consists of fused pyrimidine and imidazole rings. This system may co-exist as four N–H tautomeric forms depending on the pH [1]. Their nucleotide forms of adenine and guanine (purine base bonded through their N9 at the 2’ position of a deoxyribose ring, which is also phosphorylated at its 5’ position) (Fig. 3) are fundamental pieces for DNA and thus constitute the centers where different mutations may occur. Tautomerism of purine bases in DNA is one of the earliest recognized mechanisms to rationalize mutations and the interconversion of the different adenine or guanine tautomers (amino-imino and lactam-lactim forms) produces mispairing with pyrimidines (which also show tautomeric forms) (Fig. 4), which may lead to changes in the DNA sequence, as usually happens in some types of cancer [84–86].

**Fig. 5:**
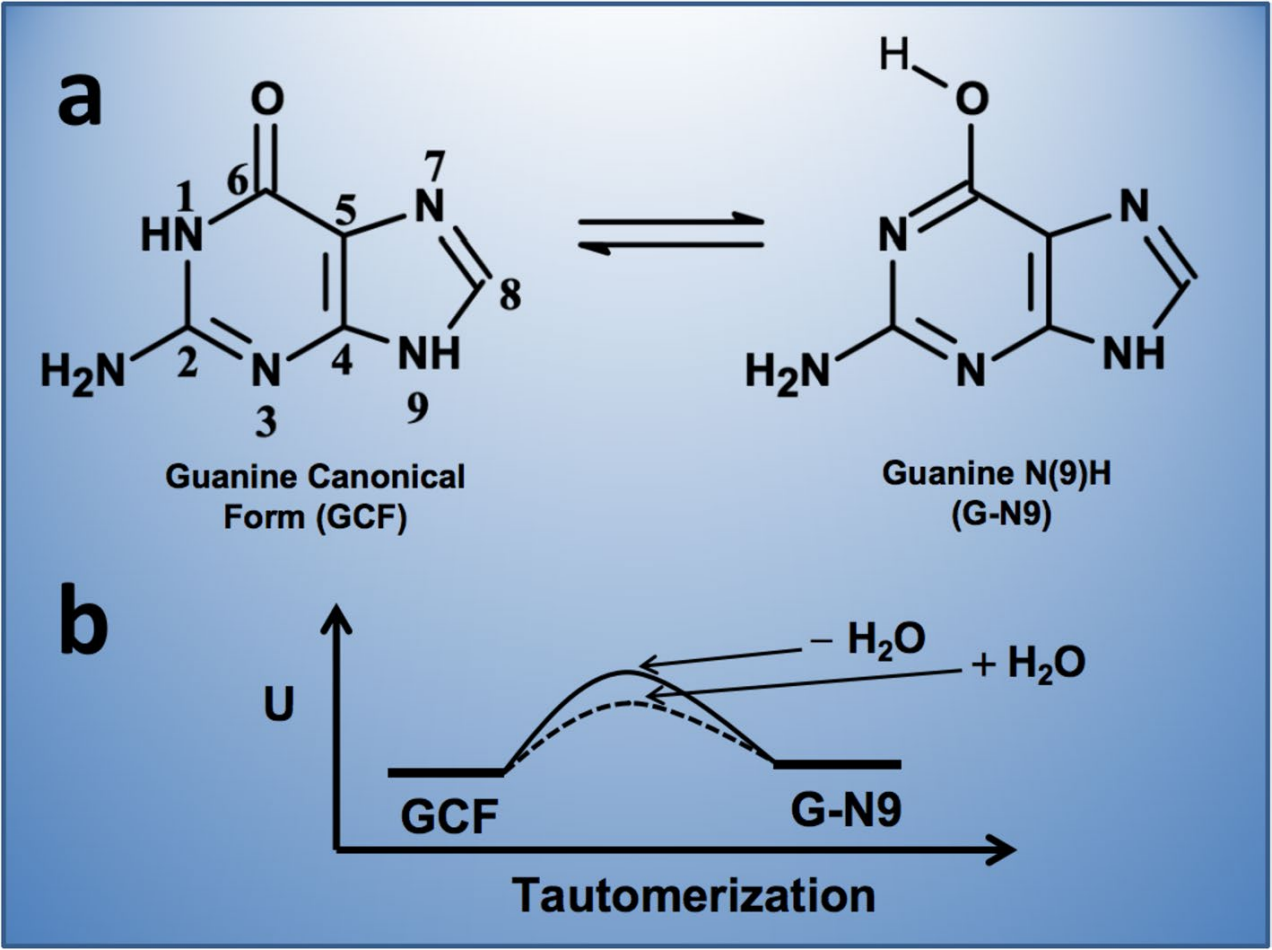
Guanine canonical and N(9)H tautomers. Solvation significantly lowers the tautomerization energy barrier to interconvert the canonical form into a N(9)H tautomer. In the absence of water, this barrier increases and tautomerization slows down

Particularly, understanding the interconversion of guanine tautomers is important because such purine is the most frequently involved purine in the process of genetic mutations [87]. Tautomers result from transitions of keto to enol forms (amine to imine in the case of adenine) [5, 6] and the consequent redistribution of hydrogen atoms in the purine molecule, so the presence of water molecules and pH are relevant in this process. In the gas phase, guanine shows three tautomers: the canonical (keto), the N(9)H (enol) and the hydroxo-amino N(7)H. Quantum chemical calculations showed that the last two forms are energetically similar to the N(9)H tautomer but, in water, the canonical form is predominant. Solvation significantly lowers the tautomerization energy barrier to interconvert the canonical form into the N(9)H tautomer, so in the absence of water, the barrier increases and tautomerization slows down [88] (Fig. 5). Within DNA the canonical form pairs with cytosine, but the N(9)H tautomer pairs preferentially with thymine; then when the DNA strand is opened, thymine commonly pairs with adenine again. It seems that when DNA is winded, the predominant interactions are hydrogen bonds between base pairs, as well as π-π stacking interactions between nitrogen bases, consequently, no water molecules exist in the interior of the double helix so the canonical form of guanine predominates and mutation rates are much lower. Once the DNA unwinds (e.g., for replication), water molecules lower the energetic barriers for tautomeric interconversion, leading to the activation of mechanisms (e.g., proofreading and base excision repair) that correct sequence changes [89, 90].

Since guanine has a low vertical ionization potential (VIP) [91], it facilitates the abstraction of one electron (e.g., induced by endogenous or exogenous radical species like those produced under oxidative stress) [92], rendering a guanine radical (especially when guanine is flanked by other guanines at 3’ and/or 5’ directions) and creating an electron hole that may lead to single base substitutions. The electron hole may migrate reversibly to various competing sites along the DNA sequence. In many cancer types, the number of mutated motifs correlates with both the free energies of base-stacking interactions and the VIPs from the target guanines. Similar correlations were found for the pathological missense and nonsense germline mutations, but only for guanines located on the non-active DNA strand [93].

N7 of guanine is the most nucleophilic atom within DNA, so many alkylating anticancer agents and mutagens (e.g., mustards, azinomycins, leinamycin styrene oxide and aflatoxin B_1_) may interact at this position. In fact, guanine N7 alkylation changes the hydrogen bonding patterns of guanine when it is paired with deoxythymidine or deoxyadenine. It has also been suggested that N7 alkylation may alter the base pairing patterns of guanine by promoting the formation of the rare enol tautomeric form of guanine [94].

Besides the tautomerism of the guanine base, its oxidation into 8-Oxo-Guanine is also an important source of mutation and carcinogenesis. 8-Oxo-Guanine is obtained through the action of the reactive oxygen species (ROS) (superoxide, hydrogen peroxide and hydroxyl radicals) mostly generated by electrons leaked from the respiratory chain in the mitochondria and other biochemical reactions. 8-Oxo-Guanine is considered a major cause for spontaneous mutagenesis and carcinogenesis, and its accumulation in the DNA increases the occurrence of A:T to C:G or G:C to T:A transversion mutations. Two different pathways induce the presence of 8-OxoGuanine in the DNA strand: the incorporation of 8-oxo-dGTP into DNA during its synthesis and the direct oxidation of the guanine base within DNA [95–97].

### Purines signaling, hepatocarcinoma and other cancers

**Fig. 6:**
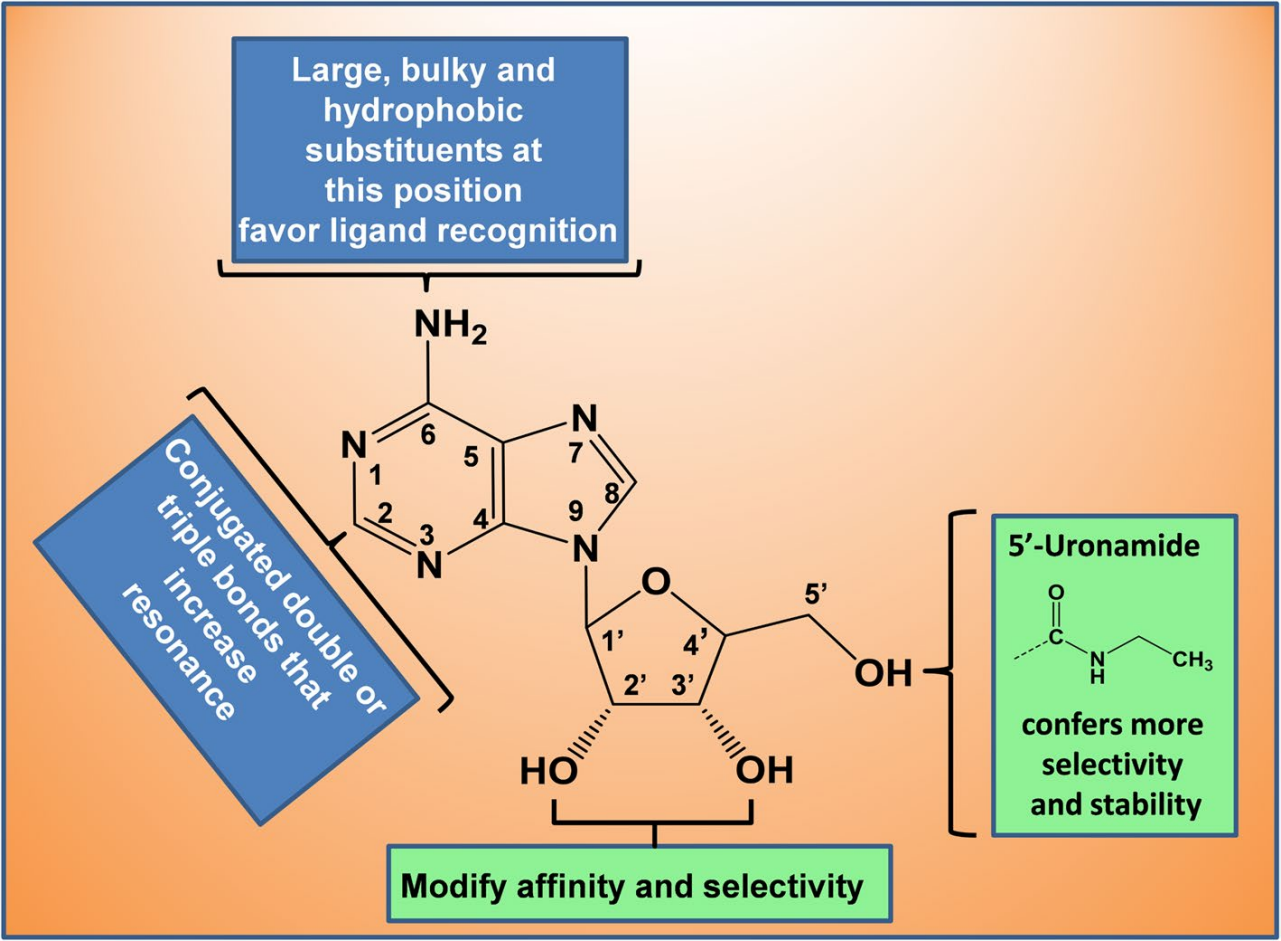
Relevance of the different ADO chemical substitutions for ADORA modulation

The formation of a malignant tumor induces hypoxia which triggers a wide network of metabolic and immunological changes that favor tumor growth and progression [98, 99], therefore, oxygen deprivation limits the availability of energy sources that brings the accumulation of extracellular ATP promoting antitumor immunity through a variety of P2X and P2Y receptors [100–102], which is subsequently degraded (by ecto-nucleotidases CD39 and CD73) to ADO that (through ADORA2A and ADORA2B) may induce cAMP-mediated downstream signaling (allowing tumor survival by immunosuppression) [103] or may inhibit cAMP generation via the activation of ADORA1 and ADORA3 for an antitumor immune response [104, 105]. Therefore, purine receptors represent an interesting target to design novel anticancer derivatives [25]. Some anticancer derivatives (e.g., 6-Chloropurine and 2-acetamido-6-chloropurine linked to a perbenzylated hexosyl moiety) have been designed with the purine ring system as a structural mother frame [106], and others have been designed specifically with adenine (in its nucleoside or nucleotide form) as a mother frame, due to the physiological relevance of adenosine receptors on many pathological processes, such as cancer, and cancer-related pain [107, 108]. So far, numerous derivatives have been synthetized and are still under experimental or clinical testing. The analysis of key structure–activity aspects of adenosine derivatives [109–111] allow us to identify some structural considerations (summarized in Fig. 6) that may provide a general understanding about the effect that chemical substitutions have on the basic structure of purines:

- 1) Adenine and ribose (from ADO molecule) are associated with regions that have different roles in the binding site(s), while adenine and other flat purines may be antagonists for ADORAs, adding a ribose moiety to the purine base confers it an adequate conformation to behave as an agonist. Thus, with proper manipulation of groups at adenine-(exocyclic) N^6^, –C2 and/or ribose moieties, a high affinity agonist may be converted into a selective antagonist or upside-down. For example, alkyl-xanthine derivatives (like caffeine) are ADORA antagonists but when linked to a ribose moiety, they act as moderately selective agonists [112].
- 2) Large, bulky and hydrophobic substituents attached at the exocyclic N^6^ (amino) of ADO favor recognition of the ligand in the binding site because of the many hydrophobic interactions around the adenine base first at the *meta*-binding site (located at the extracellular loop 3, EL3) and then at the orthosteric binding pocket, where adenine also faces π-π stacking interactions with Phe168 (EL2) [113, 114]. The presence of the exocyclic –N^6^H group in the adenine ring system increases its energetic sensitivity to structural changes [115], so its modification enhances binding recognition of ADO without altering the aromaticity of the ring system necessary for ADORA activation. Then, according to how substituents attached at the N^6^ of ADO are modified, the selectivity, affinity and potency of the ligand can also be modulated. For instance, N^6^-benzyl or N^6^-phenyladenosine are more selective than N^6^-(2-phenylethyl)adenosine, but a substitution at the 3 position of the N^6^-benzyl ring increases the affinity and selectivity. The selectivity of the ligand can be raised maximally when the benzyl group is attached to the N^6^ of adenosine through a larger (cyclic) group and also has electro-attractive atoms (e.g., CF_3_) at the 4 (*para*) position. Apparently, this *p*-CF_3_ substituent is a requisite (independently of the large cyclic group) to exhibit cytotoxicity against liver, breast and colon carcinoma cells [116].
- 3) The substitution at the –C2 position of the adenine ring system may increase potency and, in some cases, may also induce selectivity: 2-Chloroadenosine is more potent than adenosine, and if this 2-chloride atom is changed by iodine, the selectivity is enhanced. –C2 substitution combined with N^6^-Adenosine modifications can make a ligand act as an agonist or antagonist. For example, a cyano group at the –C2 in the N^6^-methyladenosine makes it an agonist, in contrast with the antagonism exhibited when the 2-Cyano group is combined with N^6^-(3iodobenzyl)-adenosine. However, if the substituent has a large and hydrophobic carbon chain but without conjugated double or triple bonds, the agonist effect is reduced. On the contrary, when the substituent increments the resonance of the adenine ring system by augmenting the electron delocalization surface, the agonist action is favored. Such resonance increment likely contributes to better ligand stability and accommodation, and recognition in the orthosteric pocket, since the –C2 position only faces an isoleucine, so there are no electronic π-π interactions to accomplish [113]. This is more evident because hydrophobic alkynyl groups enhance selectivity, and the addition of alkynyl groups at the 2 position preserves selectivity more effectively than similar chains at the 3 position (where only a water molecule bond is faced), of the N^6^-benzyladenosine [113].
- 4) Ribose moiety anchors and stabilizes the adenine base to the binding site: first at the *meta*-binding site, engaging the ADO to EL3 through hydrogen bonds and anticipating a change in orientation of the nucleoside due to hydrophobic interactions with the adenine ring system; and then at the orthosteric pocket, where ribose moiety makes and breaks different hydrogen bonds that are fundamental for the stability and selectivity of the ligand and allow up and down flips that provide the required ligand flexibility during receptor activation [114]. Substitutions at the 2’ and 3’ hydroxyl groups (2’ is more significant because it has one more hydrogen bond to face than 3’) may modify the affinity and selectivity of the ligand. If carbon or sulfur substitutes the oxygen of the ribose ring, the potency may be increased. Introducing an uronamide group at the 5’ position is a significant modification that confers more selectivity and stability for ligand recognition, since two more hydrogen bonds are registered through uronamide oxygen and nitrogen atoms [113].

As it was mentioned before, these structural considerations only pretend to give a general approach to how chemical modifications of purine derivatives affect the activity of ADORAs. However, a wider and deeper view of how such chemical modifications affect the interactions of purine derivatives (and some other compounds) with the ADORAS and how such information has had repercussions on drug design, can be found extensively on scientific literature [69, 117–119].

Hepatocellular carcinoma (HCC) is one of the most common types of primary tumors in the liver and the fourth most frequent cancer worldwide [120], and is usually the culmination of previous chronic disorders, such as cirrhosis (that comes from liver inflammation and posterior fibrosis), mostly associated to prolonged alcohol intake or viral hepatitis [22, 121]. In HCC, as in all cancers, there is an enhanced request of metabolic factors that ensure the cellular demands for biosynthetic pathways. This demand is satisfied by incrementing the uptake of glutamine (a main nitrogen donor in the biosynthesis of purines and pyrimidines as well as a cataplerotic factor), which is stimulated transcriptionally by c-myc and E2F. The c-myc oncogene, together with mutants of p53 alleles, also upregulates the expression of various purine biosynthetic enzymes, so the glutamine uptake and its utilization are facilitated in purine and pyrimidine biogenesis [122]. Liver cancer starts with inflammation and fibrosis that eventually result in liver cirrhosis with dysplastic foci that transform into low- or high-grade dysplastic nodules, which potentially become liver cancerous processes. The molecular changes in HCC include [123–125]: 1) Gene mutations caused by gene rearrangements or structural alterations (amplification or deletion) of the genome, the integration of hepatitis B virus (HBV) into the host genome, telomere shortening and TP53 pathway mutations. 2) Epigenetic modifications (dysregulated methylation, histone modifications, chromatin remodeling, miRNAs, long non-coding RNAs). 3) Alterations in signaling pathways (WNT/β-catenin, receptor tyrosine kinase, VEGF, TGFβ, JAK/STAT and ubiquitin proteasome pathways). 4) Immune escaping mechanisms. Many of these alterations are also present in other types of tumors in the liver; for example, intrahepatic cholangiocarcinoma, which is a peripheral tumor of interlobular bile ducts [126]. 5) The emergence of bioenergetic adaptation in the form of aerobic glycolysis (Warburg effect) that favors the cytoplasmic synthesis of ATP and a mitochondrial role as source of biosynthetic intermediates [127, 128].

It has been found that a crucial purine for the development of HCC is SAM, a chemoprotective methyl donor against liver cancer. Reduction in SAM levels may induce methylation deficiency and therefore genomic instability, which results in hepatocarcinogenesis [129, 130]. SAM is synthetized from L-methionine and ATP by a reaction catalyzed by adenosyl transferase isoenzymes coded by two genes: MAT1A, which is expressed in adult liver, and MAT2A, which is only present in the liver at the fetal period and is progressively replaced by MAT1A during development. MAT1A downregulation is associated with methylation of CCGG sequences in its promoter and MAT2A upregulation in human HCC is associated with CCGG hypomethylation of the gene promoter [131]. The MAT1A:MAT2A expression ratio has been directly correlated with HCC and overall DNA methylation: A reduced ratio is a prognostic marker of more malignant and lower survival HCCs [132]. MAT genes are regulated at different levels (transcriptional, post-transcriptional, and post-translational) by many dysregulated mechanisms in HCC [133]. SAM is metabolized to produce SAH, which competitively inhibits transmethylation reactions, so it must be quickly removed by the corresponding hydrolase. The SAM/SAH ratio is a liver injury indicator; low SAM and high SAH levels are characteristic of liver cancer [134]. The reduced levels of SAM are also associated with the enhanced polyamine biosynthesis that occurs in liver cancer [135], since SAM is the aminopropyl donor in that pathway that is also dependent on arginine metabolism. It has been demonstrated that treatment with an adenosine aspartyl-derivative (IFC305) increases the MAT1A and decreases MAT2A levels, restoring transmethylation activities and facilitating chemoprotective effects against HCC [136]. These chemoprotective properties have been recently reported for colon cancer too [137]. Another purine whose abnormal levels contributes to tumor pathogenesis and progression is N6-Methyladenosine which is produced by the action of methyltransferases and removed by demethylases [138].

### Immunosuppressive action of ADO in cancer generation

Ecto-nucleotidases are mediators of ADO accumulation in the cellular microenvironment. CD39 is the NTPDase1 and promotes ATP conversion to ADP and/or AMP, whereas CD73 dephosphorylates AMP to ADO [139]. Besides, activation of CD73 on tumor cells favors cell adhesion potentially through epidermal growth factor. The accumulation of ADO stimulates tumor growth and metastasis through the binding to ADORAs inducing the downstream signaling of the intracellular cAMP through ADORA2A and ADORA2B, which is associated with immunosuppression [14]. However, the activation of ADORA1 and ADORA3 inhibits cAMP generation, making them immune-promoting ADORAs [105]. Particularly, ADORA2B presents low-affinity for ADO, thus it is only activated when ADO is accumulated under pathological conditions. By coupling ADORA2B to G proteins and through downstream signaling: Gs (via cAMP) Gi (via PI3K) and Gq (via PLC), this receptor promotes different functions in the tumor microenvironment (cell proliferation, angiogenesis, immune suppression, cell invasion and inflammation) [140, 141].

### Anti-proliferative effect of ADO in blood and neural cells

As mentioned above, the accumulation of ADO contributes to tumor progression, which could be linked to T cell effector function impairment, and ADORAs could modulate human peripheral and tumor-infiltrating lymphocyte (TIL) function. It has been proposed that the ADORA2A/PKA/mTORC1 pathway impairs the immune competence of peripheral T cells and TILs, which highlights the relevance of the adenosinergic pathway as a target for immunotherapy [142]. On the other hand, stress situations (e.g. neurodegenerative diseases or hypoxia induced by brain tumor) can induce the increment of ADO concentration in the brain up to micromolar levels. This increment may induce the reversion of the reaction catalyzed by SAH-hydrolase allowing SAM accumulation and the inhibition of SAM-dependent methyltransferases. A combined receptor-mediated action of ADO and homocysteine decreased human astrocyte proliferation, mainly by a mechanism that may involve the reversal of SAH hydrolase-catalyzed reaction [143]. These findings indicate the existence of dependent and independent ADORA actions on cell proliferation.

## Conclusions

The biological role of purines is thoroughly extended in the physiopathology of all cellular systems. This role is sustained by their structural/chemical properties that make them fundamental molecules to promote mutations (e.g., the guanine base in DNA) or be the chemical donors (e.g., of methyl groups) that modulate specific biological processes. The methylation capacity (e.g., as shown by SAM) of purines is metabolically regulated and acts as a chemoprotection against cancer, so a decrement in methylation levels may induce methylation deficiency and therefore genomic instability that favors carcinogenesis. Besides, the metabolic network of purines maintains the cellular bioenergetics according to the proportion of phosphorylated and non-phosphorylated purines (from ATP to ADO, with ADP and AMP as mediators). Particularly, the intracellular homeostasis of ADO constitutes a key regulation point that influences other physiological actions that include modulation of the immune response, the regulation of catabolic and anabolic metabolism, calcium dynamics and alcohol hepatotoxicity. Extracellularly, ADO can also exert its function through the activation of ADORAs, or it can be transported into the cells by the nucleoside transporter. These receptor-mediated and metabolic properties of ADO allow diverse pharmacological actions of the nucleoside, which can seem contradictory under certain circumstances.

Purines also represent a structural base that allows the design of molecules with controlled effects. The chemical modification of such structural base reverberates on the effect on the ADORAS and/or their binding affinity by those receptors, then the ADORAS (and so the cell signals they modulate) respond according to purine concentrations and/or structural/chemical properties of the purine-derivative.

On the other hand, purinergic signaling fulfills extracellular and intracellular functions as chemical messengers and effectors with autocrine, paracrine, endocrine and metabolic actions that regulate physiological and pathological phenomena including cell metabolism, immunosuppression in tumor progression or immune promotion (depending on which receptors are involved in the cell signal) against a tumor. Particularly, the effect of ADO on the ADORAS has repercussion in many physiological events that goes from metabolic regulation to immunosuppression (cancer generation) and anti-proliferative (in neural cells) actions, passing through antioxidant and anti-inflammatory properties as well as a participant in the energy homeostasis in reproduction.

According this information, the adequate function of purine physiology is crucial for cell life and health, so when there is a homeostatic imbalance, the cell physiology is altered and its survival is compromised.

## Data Availability

Not applicable.
